# Supplementary material for: BactMAP: An R package for integrating, analyzing and visualizing bacterial microscopy data
Source: Mol Microbiol. 2019 Nov 24;113(1):297–308. doi: 10.1111/mmi.14417 (PMC7027861; doi:10.1111/mmi.14417)

Supplemental Material for

BactMAP: an R package for integrating, analyzing and visualizing bacterial microscopy data

Renske van Raaphorst, Morten Kjos & Jan-Willem Veening

Supplementary Figure S1.

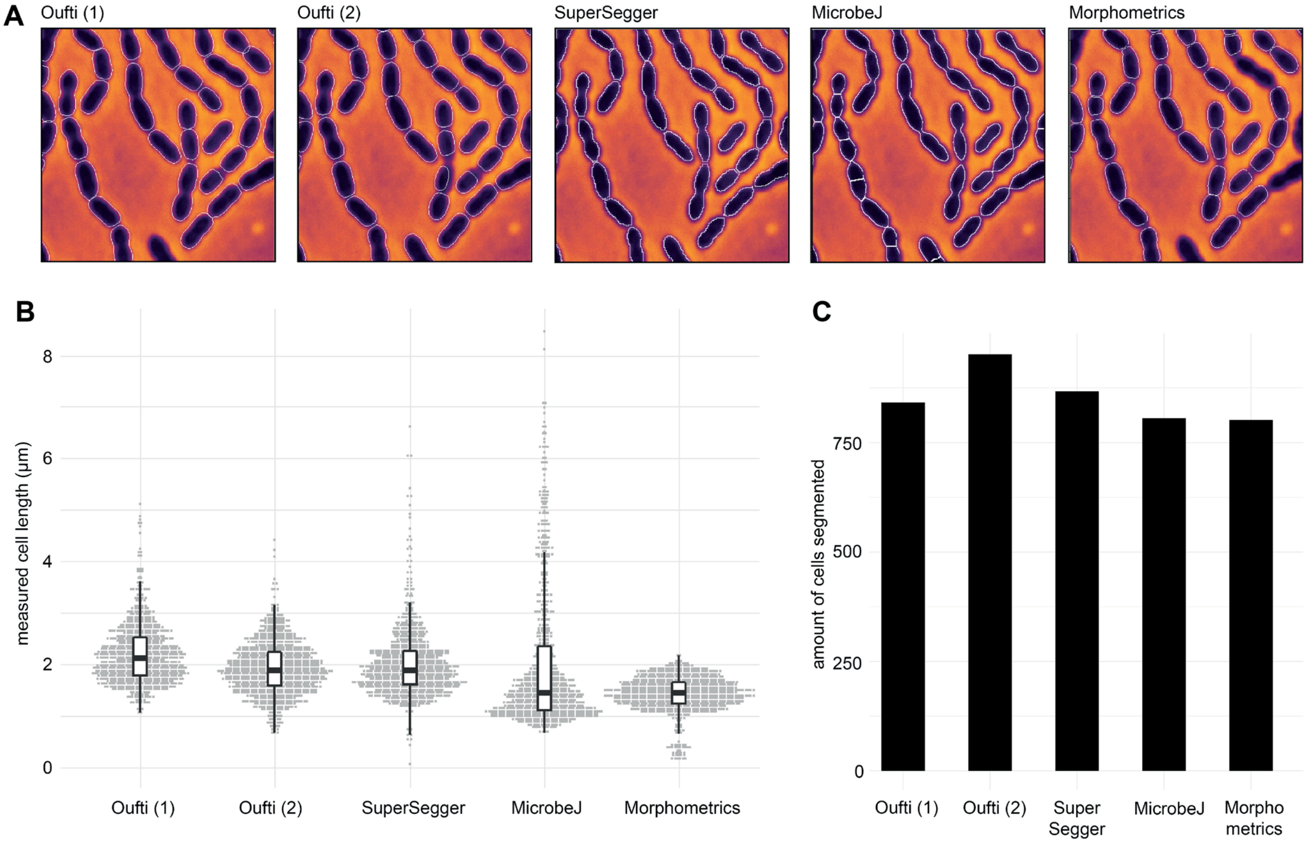

Figure S1. Comparison of phase-contrast segmentation of *Streptococcus pneumoniae* cells by Oufti, Supersegger, MicrobeJ & Morphometrics. Four lab members carried out cell segmentation of the same phase-contrast picture using the program they regularly use. One lab member decided to segment the image twice, one time using Oufti (Oufti (2) in the Fig. above) and one time using Supersegger. **A.** Cell outlines of a cutout of a microscopy image of chained *S. pneumoniae D39* cells (400*400 pixels or 20*20 µm) as segmented using the different programs. **B.** The measured cell sizes of all cells measured by each program. C: The amount of cells detected by each program.

Supplementary Figure S2.

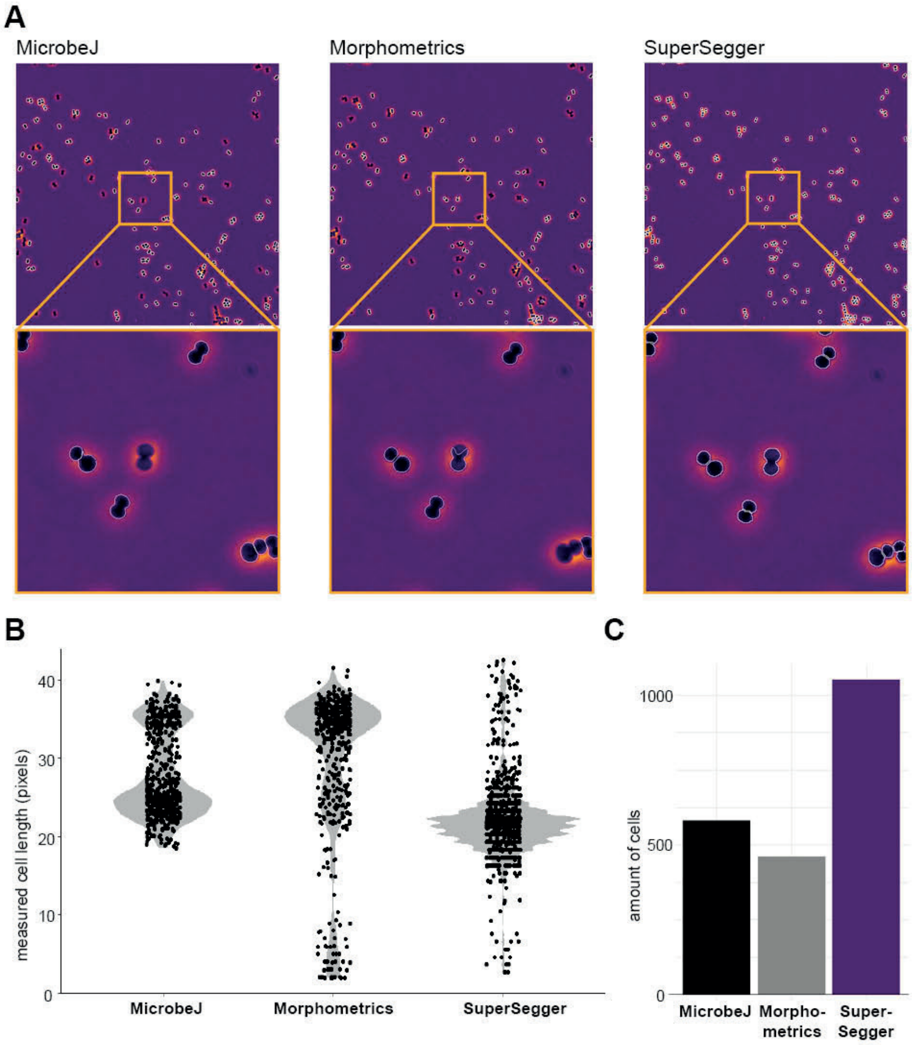

Figure S2. Comparison of *Staphylococcus aureus* segmentation using MicrobeJ, Morphometrics and SuperSegger. A. Plot of full phase-contrast image of *S. aureus* (2048*2048 px² or 113*113 μm²) with cell outlines obtained with either MicrobeJ, Morphometrics or SuperSegger (left-right) with zoomed-in cutouts below (300*300 px² or 19.5*19.5 μm²). **B.** Cell length distribution (in pixels) as measured by MicrobeJ, Morphometrics and SuperSegger, respectively. **C.** The total amount of cells found by MicrobeJ, Morphometrics and SuperSegger.

Supplementary Figure S3.

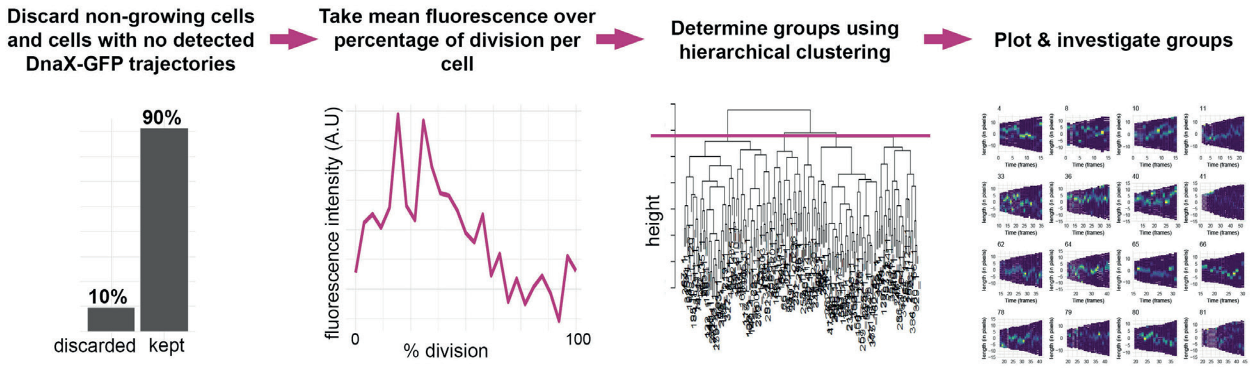

Supplement: Supplementary file 1 [file MMI-113-297-s001.pdf]
